# Abiotic Stress Response of Near-Isogenic Spring Durum Wheat Lines under Different Sowing Densities

**DOI:** 10.3390/ijms22042053

**Published:** 2021-02-19

**Authors:** Judit Bányai, Marco Maccaferri, László Láng, Marianna Mayer, Viola Tóth, Mónika Cséplő, Magda Pál, Klára Mészáros, Gyula Vida

**Affiliations:** 1 Centre for Agricultural Research, Agricultural Institute, ELKH, 2462 Martonvásár, Hungary; langlmv@gmail.com (L.L.); marco.maccaferri@unibo.it (M.M.); toth.viola@atk.hu (V.T.); cseplo.monika@atk.hu (M.C.); pal.magda@atk.hu (M.P.); meszaros.klara@atk.hu (K.M.); vida.gyula@atk.hu (G.V.); 2 Department of Agricultural and Food Sciences, University of Bologna, 40126 Bologna, Italy; mayer.marianna@atk.hu

**Keywords:** drought stress, near-isogenic spring durum wheat lines, photosynthetic activity, plant densities, yield

## Abstract

A detailed study was made of changes in the plant development, morphology, physiology and yield biology of near-isogenic lines of spring durum wheat sown in the field with different plant densities in two consecutive years (2013–2014). An analysis was made of the drought tolerance of isogenic lines selected for yield QTLs (*QYld.idw-2B* and *QYld.idw-3B*), and the presence of QTL effects was examined in spring sowings. Comparisons were made of the traits of the isogenic pairs *QYld.idw-3B++* and *QYld.idw-3B*−− both within and between the pairs. Changes in the polyamine content, antioxidant enzyme activity, chlorophyll content of the flag leaf and the normalized difference vegetation index (NDVI) of the plot were monitored in response to drought stress, and the relationship between these components and the yield was analyzed. In the case of moderate stress, differences between the NIL++ and NIL−− pairs appeared in the early dough stage, indicating that the *QYld.idw-3B++* QTL region was able to maintain photosynthetic activity for a longer period, resulting in greater grain number and grain weight at the end of the growing period. The chlorophyll content of the flag leaf in phenophases Z77 and Z83 was significantly correlated with the grain number and grain weight of the main spike. The grain yield was greatly influenced by the treatment, while the genotype had a significant effect on the thousand-kernel weight and on the grain number and grain weight of the main spike. When the lines were compared in the non-irrigated treatment, significantly more grains and significantly higher grain weight were observed in the main spike in NIL++ lines, confirming the theory that the higher yields of the *QYld.idw-3B++* lines when sown in spring and exposed to drought stress could be attributed to the positive effect of the “Kofa” QTL on chromosome 3B.

## 1. Introduction

In regions with a continental climate prone to drought, adaptability, drought tolerance and yield potential are closely related traits. The maintenance and improvement of productivity therefore require the breeding of drought-tolerant cultivars with better water-use efficiency and adaptability in changing environments and the more widespread introduction of water-conserving agricultural systems [1].

The temporal distribution of rainfall and the quantity of available water exhibit periodical changes not only at a given geographical location, but also within the crop growing season. Short periods of water deficit may occur at any time during the vegetation period, exerting varying effects on the plants, and even a mild level of water deficit may lead to severe drought symptoms if it is prolonged. The unforeseeable appearance of drought exerts an effect on yield levels: genotypes adapted to drought in one phenophase may be unable to tolerate water deficit in another phenophase [2]. The complexity of drought stress is aggravated by the fact that it may be caused by many phenomena, which also influence its severity. These include water deficit, low atmospheric moisture content, intense radiation, heat shock and high salt concentration, all of which interact with the soil type [3,4]. The combined occurrence of two different types of abiotic stress, such as drought and heat, may have an additive effect, thus causing much greater damage [5,6]. Water deficit in plants induces physiological and biochemical responses which depend not only on the intensity and duration of the stress, but also on the developmental stage of the plant and the genetically determined stress tolerance [7,8]. A decline in soil moisture content is first sensed by the root hairs, which then pass this information on to the shoots via a signal transduction system (a rise in abscisic acid content). The first detectable symptom is a loss of hydrostatic pressure (turgor) in the cells, as a consequence of which the plants close their stomata, leading to a cessation of tissue tension, cell wall ductility and growth [9]. The reduced light fixation and photosynthetic activity result in smaller biomass [10,11], lower plant height [12,13], smaller leaf area [14], fewer leaves per plant, smaller leaves and shorter leaf duration [15]. Tardieau et al. (2000) reported a shortening of the elongation zone of maize leaf tissue in response to poor water supplies and reduced transpiration, leading to a drop in the relative elongation rate [16]. Xiong et al. (2006) recorded restricted cell division in the lateral roots of *Arabidopsis* plants, with greater primary root growth [17]. Similar results were recorded by Sharp et al. (1994) in maize [18]. Lower chlorophyll content leads to earlier senescence, and this aging process is accompanied by the gradual cessation of respiration and photosynthetic processes, especially that of the sensitive photosystem II (PSII) photochemistry [19]. In the course of drought stress the photosynthetic organelles of the plant cells (the chloroplasts) are damaged by the formation of reactive oxygen species (ROS) [20]. The collapse of the photosynthetic apparatus leads to a reduction in the capacity and efficiency of photosynthetic energy storage [21,22]. Leaf senescence is a regulated process leading to the transfer of nutrients from older into younger leaves, and finally into the flag leaf. Photosynthesis in the flag leaf produces 30–50% of the assimilates required for grain filling, so this organ makes the greatest contribution to the supply of nutrients and photo-assimilates [23]. Both precocious and greatly delayed senescence may cause disturbances in nutrient remobilization [22,24], thus influencing the final yield [25]. As the result of more rapid senescence, Yang and co-workers (2000, 2001) observed increased nutrient mobilization from the leaves in wheat and rice cultivars, resulting in a shorter grain-filling period and smaller grain weight [26,27]. Many authors have reported that early and/or efficient nutrient remobilization is correlated with higher contents of protein and useful microelements—such as iron and zinc—in the grain [28].

The oxidative stress occurring in the course of water deficit and aging processes leads to the peroxidation of membrane lipids and the degradation of the ribulose-1-5-bisphosphate-carboxylase/oxygenase enzyme (RubP/carboxylase or Rubisco) in the chloroplasts due to the substantial increase in reactive oxygen species (e.g., oxygen-centered free radicals, H_2_O_2_) [29]. Rubisco is one of the first enzymes to be degraded, with a negative influence on plant photosynthesis and on the nitrogen metabolism [30]. Toxic metabolites (13-hydroperoxylinoleic acid, malondialdehyde, 4-hydroxyalkenals) are produced in the course of peroxidation, causing not only severe yield losses but also, in some cases, the death of the plant [31].

Drought stress during the reproductive phase of plant development, especially during meiosis and flowering, may result in yield losses as high as 40–70% [32]. Intact pollen grains and ovaries, an undisturbed double fertilization process, and the optimum development of the embryo and endosperm are all required for grain yield formation. The carbohydrate metabolism is disturbed by drought stress, resulting in poor development of the inner layer of the pollen wall (*intine*) and the reduced accumulation of reserve nutrients in the cytoplasm of the vegetative cells in the pollen, leading to poor attachment of the pollen on the surface of stigma papilla cells and weak pollen tube growth [33]. In experiments conducted by Jäger et al. (2013), water withholding reduced anther size in the drought-sensitive winter wheat cultivar Cappelle Desprez, with varying pollen sizes within a single flower [34]. In addition, the development rate of some pollen grains was retarded compared to the control, resulting in the formation of sterile pollen grains incapable of accumulating starch. When drought was accompanied by heat stress, the development of abnormal pollen forms was also observed. Water deficit also exerts a negative effect on the elongation of tissue cells in the ovary after fertilization and on the rapid division of cell nuclei in the endosperm. The consequent reduction in the number of endosperm cells leads to lower yields [35,36].

The values of yield components such as spike number per square meter, grain number per spike, number of fertile spikelets, thousand-grain weight and harvest index have been reported to decline as the result of physiological processes occurring in response to drought stress [12,13,37].

Creating a suitable population for examining QTL effects is a complex task because differential gene expression is caused not only by the trait of interest but also by the variation present in the genetic background [38]. One solution for establishing the functional association between the level of gene expression and a given trait is the use of a set of near-isogenic lines (NILs), which are genetically similar except for a single gene, marker or trait [39]. NILs can serve many functions, ranging from breeding purposes to the genetic analysis of complex quantitative traits. The ultimate objective of the lines determines to a large degree the choice of starting material, crossing scheme and eventually the genomic composition [40].

During the experimental period (2012/2013–2013/2014), isogenic lines of eight spring durum wheat cultivars were sown to study the effect of drought stress under field conditions using a combination of four approaches: (1) the plant responses to drought stress were tested in rain-fed and well-watered plots; (2) weather data were recorded throughout the growing period; (3) changes in the soil were monitored; and (4) the effect of the *QYld.idw-3B* QTL region on the yield was analyzed. The aim of this plant–environment–soil–genetics analysis was to give an accurate picture of the magnitude of changes in individual parameters and of the effect of drought stress on morphological, physiological, biochemical and yield biology traits. The present study was undertaken (1) to explore the correlation between morphological, physiological and biochemical parameters and yield components under drought conditions and (2) to analyze the effect of the *QYld.idw-3B* QTL region on the yield by studying near-isogenic lines of spring durum wheat with different parental alleles at the *QYld.idw* locus responsible for yielding ability on the 3B chromosome.

## 2. Results

### 2.1. Effect of Drought Stress and Plant Sowing Densities on Morphological, Physiological, Biochemical and Yield Component Traits in Near-Isogenic Lines of Spring Durum Wheat

The year, the genotype and the sowing density all had a significant effect (*p* < 0.001) on the heading, flowering and ripening dates in the field experiment, while the water supplies only had a significant influence (*p* < 0.001) on the ripening date (Table 1). In the years tested, low sowing density caused an average two-day delay in the heading, flowering and ripening dates under both irrigated and rain-fed conditions. The significant effect of the year, genotype, irrigation and sowing density could also be detected in the yield and yield components, the only exceptions being the number of sterile basal and apical spikelets in the main spike and the number of spikelets per main spike, which were not influenced by irrigation or sowing density. Low sowing density had a significant positive effect on the grain yield (t/ha), the grain weight per ear and the seed width. The seed length was significantly influenced by the year and the genotype, while irrigation had no measurable effect and low sowing density resulted in significantly longer grains in 2013 (Supplementary Materials Tables S1 and S2). When the NIL−− and NIL++ groups were compared in both field experiments the grain number, grain weight and thousand-kernel weight were found to be significantly higher for the NIL++ lines (Table 2 and Table 3).

Year and genotype had a significant effect on the chlorophyll content recorded in the Z45, Z65, Z77 and Z83 phenophases, while the significant effect of water supplies was only observed in phenophases Z77 and Z83 (Table 1). The effect of sowing density on the chlorophyll content was significant in all the phenophases tested, with the exception of flowering (Supplementary Materials Table S1). In the Z83 phenophase the chlorophyll content of NIL++ isogenic lines was significantly higher in the rain-fed treatments than that of NIL−− lines in both years. The genotype had a significant effect on the normalized difference vegetation index (NDVI) values in all three phenophases tested (Z45, Z65, Z83), while the year, water supplies and sowing density only exerted a significant effect in the Z83 phenophase. In both the rain-fed and well-watered treatments NIL++ lines had significantly higher NDVI values than NIL−−lines in the early waxy ripeness stage (Z83) (Table 1 and Table 2).

In rain-fed plots, low sowing density resulted in significantly longer stems but had no effect on the spike length or spikelet number. Great variation was observed between the individual isogenic pairs with respect to plant height. Genotype, irrigation and sowing density also had a significant effect on the plant height measured to the flag leaf and to the base and the tip of the ear. In response to water deficit there was a reduction in spike length but not in the number of spikelets. Line NIL3++ was the tallest, while lines NIL1−− and NIL1++ had the longest spikes in both treatments (Table 2 and Table 3). The relative water content of the flag leaf at flowering was significantly influenced by irrigation in both years, with 3% higher values on average in the irrigated plots. Sowing density had no effect on these values, as no significant difference in RWC was recorded within the treatment. There was no genotype effect, no significant difference being observed between the NIL++ and NIL1−− lines. The fresh aboveground biomass recorded at flowering (WW65) was not influenced by irrigation or sowing density, but the dry weight (DW65) was significantly higher in the irrigated plots in both years. No significant difference was detected between the NIL++ and NIL1−− lines. Lines NIL1++ and NIL3++ had the highest values of WW65 and DW65 in both years in both the rain-fed and well-watered plots.

**Figure 1:**
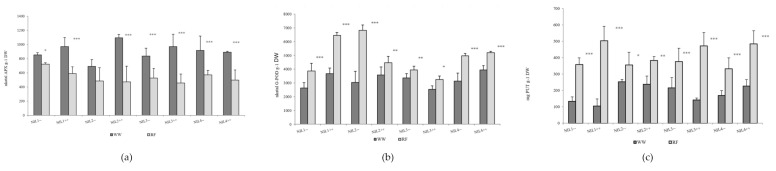
Changes in the (**a**) ascorbate peroxidase (APX) and (**b**) guaiacol peroxidase (G-POD) enzyme activity and (**c**) in the putrescine (PUT) content in the flag leaf in the well-watered (WW) and rain-fed (RF) treatments. *, ** and *** denote significant differences between the treatments at the *p* < 0.05, 0.01 and 0.001 levels of probability, respectively (2014).

The ascorbate peroxidase (APX) activity of the flag leaf declined significantly in response to drought stress in plots with normal sowing density, while the guaiacol peroxidase (G-POD) activity and putrescine (PUT)exhibited a significant rise. The reduction in APX enzyme activity was of a similar order of magnitude for all the isogenic lines (averaging 70%). Compared to the well-watered plots, water deficit caused a substantial rise in G-POD activity in the tested lines. The greatest relative increase was detected for the lines NIL1++, NIL2−− and NIL4−−. Water deficit induced the accumulation of PUT, SPD and SPM in both the NIL++ and NIL1−− lines, with the greatest change in the quantity of putrescine per unit dry matter (Figure 1). The agmatine and cadaverine contents were below the detection limit. Lower values of PUT, SPD and SPM content were recorded for lines NIL1−−, NIL1++ and NIL3++ in the irrigated plots, but in response to drought stress higher accumulation was observed for these lines than for the other isogenic lines.

### 2.2. Interaction between the Morphological, Physiological, Biochemical and Yield Component Traits in Near-Isogenic Lines of Spring Durum Wheat under Drought Stress

**Figure 2:**
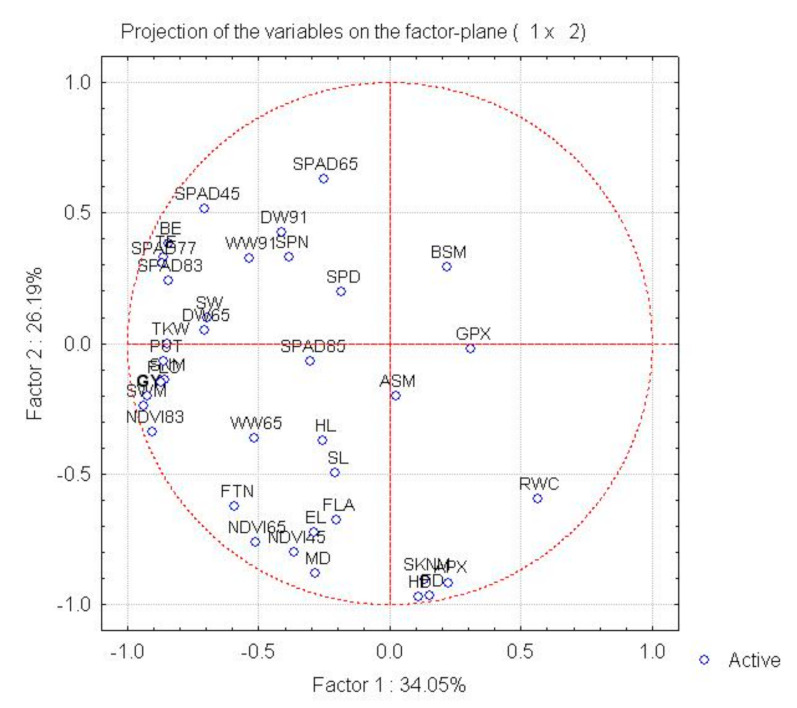
Correlation system demonstrated by principal component analysis between plant development, morphological, physiological and biochemical traits and the yield and yield components based on the parameter matrix formed from the mean values of rain-fed treatments over two years.

Principal component analysis on correlations between the traits revealed six factors with eigenvalues greater than unity, which together explained 97.89% of the total variance. The first background variable (34.05% of total variance) was most closely correlated with the yield (r = 0.92 ***), and also with various yield components (SNM, SWM, TGW, SW; r = 0.70 **−0.93 ***), with morphological (FLC, BE, TE, DW65; r = 0.84 ** to −0.87 ***) and physiological parameters (SPAD45, SPAD77, SPAD83, NDVI83; r = 0.70 **–0.92 ***), and with putrescine (r = 0.86 ***). The second factor (26.19% of total variance) was correlated with the phenophases (HD, FD, MD; r = 0.88 ***−0.97 ***), morphological traits (SKNM, FTN, FLA, EL; r = 0.62 ** to −0.91 ***), physiological parameters (NCVI45, NDVI65: r = 0.76 **–0.80 **) and ascorbate peroxidase activity (r = 0.92 ***). The chlorophyll content at flowering (SPAD65; r = 0.63), the fresh biomass (WW65; r = 0.75 **), the test weight (r = 0.80 **) and the grain width (r = 0.70 **) exhibited a correlation with the third background variable. Based on the factor–variable correlation pattern, eight of the traits tested—the grain number, grain weight, seed width and thousand-kernel weight recorded for the main spike after harvest, the dry biomass at flowering, the NDVI value recorded at early waxy ripeness, the plant height to the flag leaf and the putrescine content—could be grouped with the yield (Figure 2).

**Figure 3:**
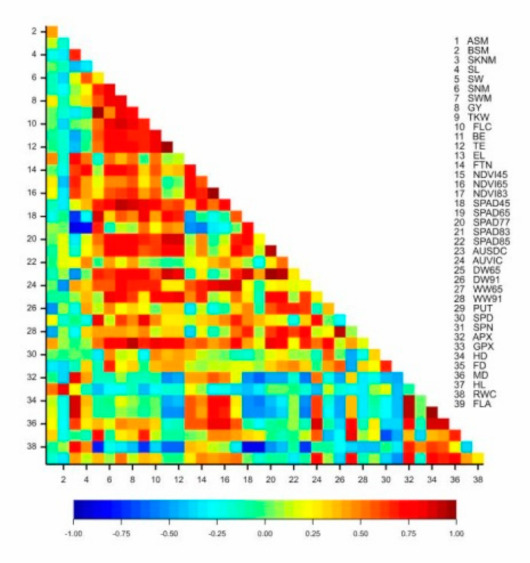
Pearson’s correlation coefficients for the plant development, morphological, physiological and biochemical traits and the yield components of eight near-isogenic lines of spring durum wheat in the rain-fed treatment (2013–2014). Critical r values for the correlation coefficients: 0.6319 (*p* < 0.05); 0.7646 (*p* < 0.01); 0.8721 (*p* < 0.001).

The yield of the isogenic lines exhibited a significant correlation with the thousand-kernel weight (r = 0.735 *), the number of grains per main spike (r = 0.846 **), the grain weight (r = 0.844 **), the height parameters (FLC: r = 0.800 *; BE: r = 0.679 *; TE: r = 0.717 *), the vegetation activity of the plots at flowering and grain filling (r = 0.684 *, 0.863 **), the chlorophyll content of the flag leaf in phenophases Z77 and Z83 (r = 0.786 **, 0.772 **), the number of side-tillers per square meter (r = 0.689 *) and the dry biomass at flowering (r = 0.648 *). The APX enzyme activity was in significant positive correlation with the number of spikelets on the main spike (r = 0.803 ***), the guaiacol peroxidase (GPX) enzyme activity with the number of sterile basal spikelets (r = 0.847 ***), the spermine content with the grain number per main spike (r = 0.658 **) and the putrescine content with the thousand-kernel weight (r = 0.664 **), the yield (r = 0.931 ***), the grain number per main spike (r = 0.734 **) and the grain weight (r = 0.780 **) (Figure 3).

The correlations demonstrated that the yield was determined most significantly by the number of side-tillers per square meter (which explained 40.41% of phenotypic variance), the grain number per main spike (48.87%) and the grain weight in the main spike (22.79%). The plant height to the flag leaf and to the tip of the ear correlated significantly with the yield and the thousand-kernel weight. The chlorophyll content of the flag leaf in the Z65, Z77, Z83 and Z85 phenophases was in significant correlation with the thousand-kernel weight, the grain weight in the main spike and the seed width. The grain weight of the main spike was also significantly influenced by the chlorophyll content in phenophases Z77 and Z83, while the number of grains per main spike was correlated with the chlorophyll content at the milk development stage and the flag leaf area. The light reflectance of the plots at the early dough stage (NDVI83) exhibited a significant correlation with the yield, the grain weight of the main spike and the grain number per main spike. The aboveground fresh and dry biomass at flowering was significantly correlated with the yield, the thousand-kernel weight, the grain weight of the main spike and the seed width (Table 4).

## 3. Discussion

Near-isogenic lines are efficient tools for investigations of the function and regulation of single genes [41,42]. The reason for using NILs is that pairs of near-isogenic lines, which have the same genetic background but differ at a single locus, can be used to turn quantitative traits into Mendelian factors, thus providing a better insight into genotypic and phenotypic interactions [43]. In the present experiment the joint effect of year, genotype, sowing density, QTL region and irrigation was investigated in the *QYld.idw-3B* QTL region on the short arm of chromosome 3B using near-isogenic lines of spring durum wheat. Low soil moisture content and high temperatures were recorded from the start of the milk development stage in both years of the field experiment. Significant differences in NDVI values between the NIL++ and NIL1−− pairs were already visible at the early dough stage (Z83), indicating that the *QYld.idw-3B++* QTL region was able to maintain photosynthetic activity for a fairly long period, resulting in higher grain numbers and grain weight at the end of the vegetation period. Naser et al. (2020) also reported that NDVI values obtained from canopy sensing could be useful for assessing the yields of individual wheat genotypes [44]. The overall agreement between NDVI values and grain yield classes for different growth stages suggested that wheat genotypes could best be differentiated and classified between anthesis and the middle of grain filling. Fu et al. (2020) constructed a multifactor model to increase accuracy and stability and demonstrated that the correlation between the vegetation index and the yield was better in later phenophases than in the early growth stage [45]. This is particularly true in the Carpathian Basin, where drought stress occurs most frequently in the grain-filling and early dough stages after the cessation of May rainfall. The chlorophyll content of the flag leaf in phenophases Z77 and Z83 was in significant correlation with the grain number and grain weight of the main spike, the thousand-kernel weight and the plant height, confirming the likelihood that correlations between vegetation indices, yield and yield components should be sought in phenophases after flowering. In the course of breeding, the physiological parameters that are in positive correlation with the yield during drought stress (the chlorophyll content of the flag leaf, remote sensing-based indices) could be suitable for the identification of genotypes with stay-green traits, which could make them more tolerant of drought [46,47].

If the soil does not contain sufficient water for plant development, growth will be inhibited and less biomass will be formed, resulting in poor grain filling. The yield will decline due to smaller grain size or, if the stress occurred in an earlier phenophase, due to a smaller number of grains per spike. Positive turgor is essential for the maintenance of growth [48], which is why higher values of plant height parameters (FLC, BE, TE) and dry biomass at flowering (DW65) were recorded for the well-watered treatment in both years [49]. The factor–variable correlation pattern showed that morphological traits related to biomass and the vegetation activity at grain filling played an important role in the development of the yield components that determine the yield. Measurements of the vegetation activity of the plots gave a good reflection of the number of side-tillers per unit area and the area of the flag leaf, as proved by the fact that these traits were put into the same group in the course of principal component analysis.

Measurements on changes in stem dry weight can be used to estimate the level of nutrient mobilization into the grains [50,51] and many authors have reported the detection of genetic variability in the storage capacity of vegetative organs and in the efficiency of remobilization. In the present work, the dry biomass exhibited a significant positive relationship with the yield and yield components (GY, SNM, SWM) and with the height parameters (FLC, BE, TE), so its genetic variability during drought stress could make it useful in breeding [52]. These results are in agreement with the findings of Korohou et al. (2020), who reported that wheat grain yield could be estimated from the total dry matter of the leaves, stem and ear. When six models developed by these authors were evaluated on a test dataset the results showed that the models were generally accurate in terms of grain yield prediction [53].

The grain yield was greatly influenced by the water supplies, while the genotype had a significant effect on the thousand-kernel weight of the main spike. When the lines were compared in the rain-fed treatment, significantly more grains and significantly higher grain weight and thousand-kernel weight were recorded in the main spike for the NIL++ lines, confirming the possibility that the higher yield of the *QYld.idw-3B++* lines in spring sowings subjected to drought stress could be attributed to the positive effect of the Kofa QTL on the 3B chromosome. Bennett et al. (2012) identified two QTL regions on chromosome 3B that exhibited a close correlation with yield and plant temperature in a doubled haploid population arising from a cross between a drought-tolerant line and a sensitive wheat cultivar (RAC875/Kukri) [54]. A Meta-QTL linked to yield [55], a QTL closely associated with plant temperature, the chlorophyll content at the milk development stage and the NDVI value during heat stress [56] were also found on this chromosome. A region correlated to yield, thousand-kernel weight and early growth vigor was identified by Bonneaue et al. (2013) in 21 different environments in a doubled haploid winter wheat population [57]. The *qGYWD.3B.1* and *qGYWD.3B.2* genomic regions detected on chromosome 3BS exhibited significant associations with yield, grain number per square meter, number of fertile side-tillers and spike number in a winter wheat RIL mapping population [58]. In a set of substitution lines with varying levels of drought tolerance, Dudziak et al. (2019) found that chromosomes 3B, 5A, 7B and 7D had the greatest impact on the initial wheat response to short-term stress and could therefore be critical for the genetic control of drought tolerance in wheat [59].

Li et al. (2016) reported a significant reduction in various yield components as the sowing density rose [60]. At low plant densities, Whaley et al. (2000) also found an increase in the relative growth rate of the crop, including a longer period of tiller production, better radiation use efficiency, greater green area per shoot and improved shoot survival, which prevented a decline in crop dry matter production [61]. The greater area available to each plant at low sowing density clearly had a significant positive effect on the grain weight and seed width per spike and on dry biomass (DW65). This was promoted by a number of indirect traits (higher chlorophyll content and NDVI value, longer stems, larger root system). Genotypes with good tillering ability are able to perform better at low sowing density, probably due to the better utilization of water, solar radiation and nitrogen [62,63]. As an early signal of cell division, light influences the development of new leaves and tillers, thus enhancing the photosynthetically active green plant area. This was confirmed by the significantly higher biomass, vegetation index and flag leaf chlorophyll content. It should not be forgotten, however, that for genotypes with poor tillering ability and a lower number of tillers, higher sowing density is required for the development of a satisfactory yield [64]. If wheat yields are to continue to improve, it is essential to define the optimum agronomic plant density, i.e., the minimum number of plants per unit area required to maximize yield [65].

Strong correlative and analytical evidence assigns a major role in drought tolerance to the redox regulatory and antioxidant system. The strong antioxidant system found in resurrection plants underlines the importance of the antioxidant system for surviving long periods of dehydration [66]. Changes in antioxidant enzyme activity during drought stress are greatly dependent on the plant species and cultivar and on the severity and duration of the stress. The wheat species investigated by Pour-Benab et al. (2019) exhibited diverse responses to drought stress, particularly as regards the ability of APX, GPX and SOD (superoxide dismutase) to scavenge reactive oxygen species [67]. In barley plants the activity of the antioxidant enzymes catalase, guaiacol peroxidase and superoxide dismutase was significantly higher in drought-stressed treatments, while the activity of ascorbate peroxidase did not change significantly with the treatment [68]. In the present experiment the reduction in the APX activity of the flag leaf in the non-irrigated treatment was of a similar magnitude in all the isogenic lines. By contrast, higher GPX activity was recorded in the flag leaf of all the lines in the non-irrigated treatment compared to plots with good water supplies. It would seem that sensitive plants tend to activate the glutathione-dependent scavenging system rather than the ascorbate-dependent system, which is only induced to a moderate extent, or may even be down-regulated [66]. Numerous yield components proved to be in negative correlation with the activity of the ascorbate peroxidase and guaiacol peroxidase enzymes and in significant positive correlation with the polyamine content (SW, SNM, SWM), thus confirming the role of these compounds in the stress response under field conditions. It is clear that increasing the activity of antioxidant enzymes is one strategy in the drought tolerance mechanisms in plants. When investigating changes in antioxidant enzyme activity throughout the growing period in wheat genotypes grown with normal or deficient water supplies, Huseynova et al. (2010) concluded that drought caused changes in the balance between free radical production and protective enzyme reactions [69]. Polyamines (PAs) are important for plant growth and development and for their environmental stress responses, thus acting as a novel plant biostimulant [70]. In the present experiment the accumulation of polyamines rose during drought stress in the flag leaves of the isogenic lines, with the greatest increases for putrescine, spermidine and spermine. A positive correlation was found between the polyamine content and the grain number/grain weight of the main spike in the rain-fed treatment. Water deficit induced the accumulation of PUT, SPD and SPM in both NIL++ and NIL1−− lines but the greatest change induced by the treatment in the detectable polyamine content was observed for the quantity of putrescine per unit dry matter. In experiments on the use of waste water to irrigate wheat, Aldesuquy et al. (2014) demonstrated that the application of spermine or spermidine was able to mitigate the negative effects of the treatment on the yield and yield components [71]. Liu et al. (2016) agreed that the interaction between hormones rather than the action of a single hormone was involved in the regulation of wheat-grain filling under drought [72]. However, although the overexpression of certain enzymes could enhance drought tolerance, it could also cause great delays in germination and development, thus disrupting the growing season [66]. Therefore, mapping strategies should be devised for the localization of genetic loci associated with the response of cereals to drought [59].

Due to the multiplicity of factors influencing experiments carried out under field conditions, there is limited scope for determining the degree of stress tolerance using a single parameter. Also, because of the substantial interaction between the plant and the environment, genotypic differences may also occur within the same plant [73]. In the future, the use of systems biology approaches such as genomics, transcriptomics, proteomics and metabolomics could result in new techniques for the identification of key regulators, genes, proteins and metabolites, which will help breeders to develop new, stress-tolerant genotypes [74].

## 4. Materials and Methods

### 4.1. Plant Materials

Eight near-isogenic lines of spring durum wheat, derived from four different recombinant inbred lines of a cross between the spring durum wheat cultivars ”Kofa” and ”Svevo” [75], were examined under rain-fed (RF) and well-watered conditions (WW) in the nursery of the Agricultural Institute at Martonvásár, Hungary, during the 2013 and 2014 spring cropping seasons.

The NIL pairs were obtained by MAS (Marker-assisted selection), selecting pairs differing only for the 3BS QTL (the NILs were all fixed for “Kofa KK allele” on chromosome 2B). When the *QYld.idw-3B* region on chromosome 3B was “Kofa KK” (KK2BLKK3BS), the NILs were coded as ++ (“NIL1++”, “NIL2++”, “NIL3++”, ”NIL4++”), and when the allele was “Svevo SS” (KK2BLSS3BS), they were coded as −− (”NIL1—“, ”NIL2−−”, ”NIL3−−”, ”NIL4−−”) [38].

### 4.2. Site Description and Set-Up for Field Experiment

**Figure A1:**
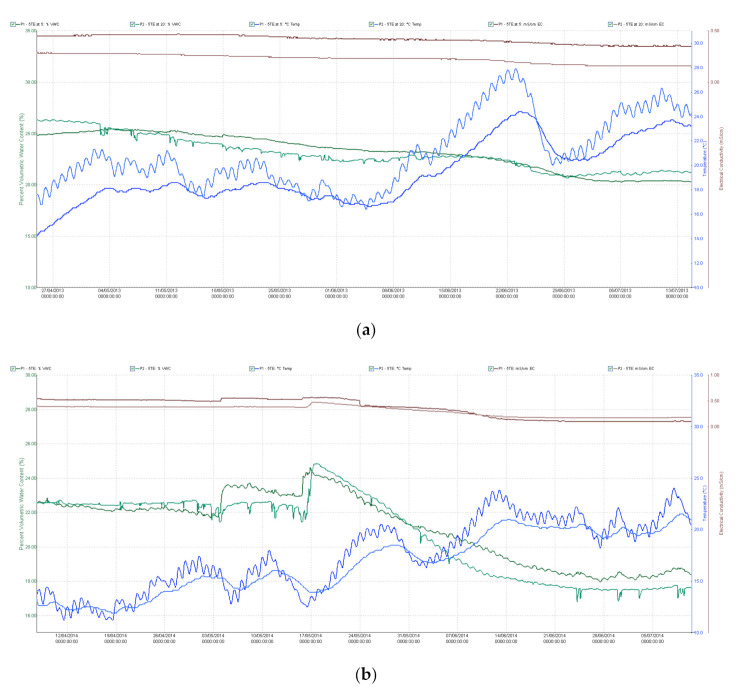
(**a**) Trends in soil moisture content, soil temperature and electrical conductivity at depths of 30 and 60 cm in the rain-fed plots (**a**) 2013 growing season; (**b**) 2014 growing season.

The experiments were laid out in an unbalanced incomplete alpha lattice block design with eight replications for rain-fed and eight for well-watered conditions under two sowing densities (480 seeds/m^2^ normal, 320 seeds/m^2^ low). The geo-coordinates of the field were latitude 47°18′39.81″ N and longitude 18°46′42.83″ E (Martonvásár, Hungary), and the soil was classified as chernozem with forest residues. The genotypes were sown in the middle of March each year. Individual plots consisted of eight rows, 12 cm apart, in 0.96 m x 4 m plots. No disease was observed during the growth period and weeds were controlled using chemicals. Capacitive soil moisture sensors (5TE—Decagon Devices, Inc., Pullman, WA, USA) were placed at depths of 30 and 60 cm at eight points, all in the plot containing the line ”NIL1++”. Measurements of the moisture content (*v*/*v* %), temperature (°C) and electrical conductivity (dS/m) of the soil were made every hour throughout the growing season (Figure A1a,b).

Based on the pF curve, the water stress state was associated with a value of 21.5 *v*/*v* % (pF 3.4). The change in tension was constantly monitored with an MPS-1 tensiometer. Values of −30 to −40 kPa represent satisfactory water supplies for the crop, while values lower than −40 kPa stress the plants and irrigation is required to ensure optimum development and yields. Irrigation was carried out by means of microirrigation with microjets (microsprinkler technology), which distributed a uniform quantity of water in the well-watered replications. An ECRN-100 rainfall meter with a sensitivity of 0.2 mm was used for the accurate measurement of the quantity of water applied. In April 2013 (the shooting period) the plants suffered from rainfall deficiency and despite rainfall in May the soil moisture content dropped below the critical 22.3 tf% level (at depths of 30 and 60 cm) after the start of the dough development stage (Z83). This period was very hot, with maximum temperatures averaging 29.7 °C in the second and third weeks of June. Irrigation started in the booting stage (Z45) and was continued regularly till the end of July to keep the soil tension around −35 kPa. In 2014 the soil moisture content began to fall after flowering (Z65), partly due to the high temperatures recorded from May 20th onwards. In the rain-fed plots, values below 22.3 tf% were measured during grain filling, demonstrating that little water was available to the plants. Water supplies were provided on five occasions during the 2014 growing season, from the boot stage (Z45) to the early dough development stage (Z83). Air temperature (min, max, mean), humidity, global radiation, wind speed and rainfall quantity were recorded daily throughout the growing season.

### 4.3. Data Collection

The phenology of each accession was evaluated on the basis of the Zadoks scale [76]. The chlorophyll content of the flag leaf was measured using a SPAD-502 chlorophyll meter (Konica Minolta Sensing, Inc., Osaka, Japan) and expressed as a relative value (SPAD value) at the boot stage (SPAD45), at flowering (SPAD65), in the late stages of milk development (SPAD77), at early dough (SPAD83) and at the end of dough development (SPAD85). Readings were taken at three positions on the flag leaf—the base, the middle and the tip of the leaf lamina—in 24 replications per line for each water regime. The average per plot was computed and used in the analysis. The NDVI was measured in three phenophases: at the boot stage (NDVI45), at flowering (NDVI65) and at early dough development (NDVI83), using a GreenSeeker-505 handheld sensor (Trimble, Inc., Sunnyvale, CA, USA). Both the mean plant height, measured to the flag leaf collar (FLC), to the base of the ear (BE) and to the tip of the ear (TE) at the end of the physiological maturity phenophase, and the ear length (EL) were determined in 12 replications. The flag leaf area (FLA) at flowering (Z65) was determined as the mean value of the flag leaf area on the main tiller of ten representative plants (taken from positions where no plants were missing) using a LI-3100C leaf area meter (LI-COR, Inc., Lincoln, NE, USA). The relative water content (RWC%) of the flag leaf was calculated at flowering (Z65) on the flag leaves on the main tiller of three representative plants. The biomass was recorded in the flowering (Z65) and physiological maturity (Z91) phenophases, based on measurements of the whole aboveground fresh (WW65; WW91) and dry (DW65; DW91) biomass of ten representative plants. The ascorbate peroxidase and guaiacol peroxidase activity and the polyamine content (PUT, SPD, SPN) were measured in the flag leaves of the main tiller in five replications on samples collected from rain-fed and well-watered plots at flowering (Z65), as described by Pál et al. (2013) [77]. The plots were harvested with a small-plot combine, and data were recorded for the yield (GY) and yield components, including the thousand-kernel weight (TGW) and the average grain width (SW) and length (SL) on the main spike, determined using the Marvin digital seed analyzer system. Measurements were made on the spikelet number per spike for 16 main spikes (SKNM) per line, the grain number and grain weight per main spike (SNM), and the number of sterile apical (ASM) and basal (BSM) spikelets per main spike.

### 4.4. Statistical Analysis

Analysis of variance on the phenotypic data was performed for each individual year and for all the years combined using the linear random/mixed effect model in the Genstat 18 software package. Interactions and correlations between the traits and treatments were determined using principal component analysis, and means within a treatment were designated by different letters (*p* = 0.05) based on Duncan’s multiple range test. A modified equation was used to calculate the area under the SPAD value decline curve and the area under the vegetation index curve, which allowed genotypes that stayed green for a longer period to be selected [78].

$$AUSDC=\overset{n-1}{\underset{i=1}{\sum }}\left[\frac{G\left(i+1\right)+G\left(i\right)}{2}\right]*\;\left[D\left(i+1\right)-D\left(i\right)\right]$$

*Gi* = SPAD value recorded on the ith day, *Di* = ith day, *n* = number of days.

$$AUVIC=\overset{n-1}{\underset{i=1}{\sum }}\left[\frac{V\left(i+1\right)+V\left(i\right)}{2}\right]*\;\left[D\left(i+1\right)-D\left(i\right)\right]$$

*Vi* = NDVI value recorded on the *i*th day, *Di* = *i*th day, *n* = number of days.

## 5. Conclusions

In the present study, a complex field phenotyping system was described which, by a joint plant–environment–soil–genetics analysis, gave an accurate picture of the magnitude of the changes occurring in individual parameters and of the changes caused by drought stress in the morphology, physiology and yield biology of the plants. Lower plant density, which resulted in a larger growing area per plant, clearly had a significant positive effect on the grain weight per spike and the grain width. This may have been promoted by a number of indirect traits (higher chlorophyll content and NDVI value, longer stems, larger root system) and shows that genotypes with good tillering ability perform better at lower plant density. This was probably also aided by the better utilization of water, solar irradiation and nitrogen. The significantly higher grain number, grain weight and thousand-kernel weight in the main spike for the NIL++ lines confirmed the possibility that the higher yield of the *QYld.idw-3B++* lines in spring sowings subjected to drought stress could be attributed to the positive effect of the Kofa QTL on the 3B chromosome. The better yield stability of lines with the KK_2BL_KK_3BS_ allele combination indicates that it is worth giving further attention to the role of the *QYld.idw-2B* and *QYld.idw-3B* QTL regions in yield enhancement in durum wheat.

## Figures and Tables

**Table 1:** Significance of year, genotype, treatment and sowing density effects and interactions on plant development, morphological and physiological parameters and on yield components, based on the Chi-squared test for eight near-isogenic lines of durum wheat. In the REML analysis, F = Wald statistic/d.f., combined over the two years. *, **, *** significant at the *p* < 0.05, 0.01 and 0.001 probability levels, respectively.

	Year (Y)	Genotype (G)	Treatment (T)	Density (D)	G × Y	G × T	G × D	Y × T	Y × D	T × D	Y × G × T	Y × G × D	Y × T × D	G × T × D	Y × G × T × D
**d.f.**	1	7	1	1	7	7	7	1	1	1	7	7	1	7	7
**HD ^1^**	2156.85 ***	502.57 ***	3.71	525.87 ***	27.06 ***	2.02	6.53 ***	6.77 *	0.08	12.71 ***	3.14*	5.26 ***	1.82	1.27	0.48
**FD ^2^**	3167.93 ***	332.91 ***	1.88	393.75 ***	20.86 ***	1.24	3.97 ***	8.72 **	6.44 *	9.22 **	2.53 *	3.78 ***	1.88	1.43	1.12
**MD ^3^**	3798.87 ***	157.56 ***	377.06 ***	119.30 ***	25.58 ***	4.84 ***	3.81 ***	21.11 ***	27.96 ***	20.32 ***	3.21 **	4.53 ***	26.16 ***	1.56	1.03
**GY ^4^**	72.11 ***	30.10 ***	247.04 ***	109.42 ***	8.23 ***	1.58	2.62 *	78.62 ***	32.88 ***	37.09 ***	2.24 *	1.24	33.14 ***	2.12 *	2.75 **
**TGW ^5^**	133.71 ***	88.35 ***	685.52 ***	56.52 ***	20.26 ***	9.06 ***	2.87 **	487.73 ***	0.08	0.52	2.46 **	0.83	1.95	2.81 **	3.02 **
**SNM ^6^**	826.46 ***	8.50 ***	2.02	0.08	1.27	0.27	1.09	0.03	2.84	0.94	0.79	0.16	0.95	1.06	0.79
**SWM ^7^**	132.34 ***	27.68 ***	414.71 ***	133.16 ***	1.76	1.42	1.73	0.71	3.46	0.19	1.03	0.95	24.78 ***	0.51	0.48
**SKNM ^8^**	644.12 ***	53.33 ***	7.97	24.60	0.45	1	9.62 **	3.14	0.38	0.04	1.39	1.43	0.10	0.55	0.98
**BSM ^9^**	1.34	0.58	0.28	0.07	0.69	0.40	0.43	0.11	0.26	0.51	0.91	0.46	0.15	0.91	0.28
**ASM ^10^**	0.37	0.59	1.17	0	0.89	0.77	0.68	1.66	0.03	0.07	0.56	0.83	0.03	0.85	0.75
**SL ^11^**	4.06 *	13.07 ***	0.68	31.23 ***	4.09 ***	1.90	1.17	0	26.32 ***	15.52 ***	4.66 ***	3.50 *	4.06 *	0.76	1.99
**SW ^12^**	600.45 ***	220.04 ***	34.51 ***	16.23 ***	44.47 ***	1.77	7.44 ***	600.45 ***	30.68 ***	28.85 ***	3.14	1.70	8.14 ***	1.38	0.99
**FLC ^13^**	876.31 ***	18.48 ***	16.41 ***	13.94 ***	12.82 ***	1.77	1.16	27.49 ***	4.27 *	31.21 ***	1.69	0.74	0.1	1.34	0.74
**BE ^14^**	978.21 ***	128.18 ***	52.24 ***	16.60 ***	32.02 ***	3.31 *	1.20	12.97 ***	0.58	41.13 ***	2.88 *	2.00	2.17	1.21	1.66
**TE ^15^**	979.14 ***	102.85 ***	62.92 ***	22.70 ***	35.24 ***	0.28	0.41	7.33 **	1.26	22.70 ***	1.18	0.55	1.31	2.02	1.37
**EL ^16^**	101.32 ***	4.38 ***	25.08 ***	8.56 ***	0.72	0.43	0.41	0.42	0.02	0.89	0.13	0.15	0.02	0.17	0.07
**FTN ^17^**	0.41	9.97 ***	0.35	7.77 **	8.19 ***	2.27 *	1.12 *	0.29	1.68	0.11	1.76	0.38	0.15	1.04	0.26
**FLA ^18^**	28.45	71.72	0	2.01	17.07	3.21	2.28	0.46	0.15	0.06	2.61	0.70	0.21	1.09	0.82
**SPAD45 ^19^**	1610.61 ***	18.52 ***	2	36.93 ***	1.86	1.75	1.21	0.25	0.20	11.43 ***	1.06	1.04	1.23	0.87	1.27
**SPAD65 ^20^**	220.84 ***	16.70 ***	0	7.10	3.14	1.02	0.66	0.11	1.11	1.83	0.65	1.06	1.40	1.44	0.46
**SPAD77 ^21^**	202.70 ***	26.52 ***	70.18 ***	28.00 *	10.75 ***	2.29 *	0.68	0.73	52.70 ***	3.25	1.91	0.18	3.79	1.14	1.38
**SPAD83 ^22^**	14.41 ***	11.60 ***	336.60 ***	19.00 *	5.23 ***	3.15 **	0.35	1.51	55.48 ***	18.87 ***	1.20	0.23	0	0.46	0.49
**SPAD85 ^23^**	2.63	3.70	23.07	19.43 ***	3.66 ***	1.20	1.03	11.20 ***	27.94 ***	0.56	1.40	0.62	10.69 **	0.21	0.96
**NDVI45 ^24^**	1.05	16.91 ***	0.02	0.02	3.82 ***	0.87	0.15	0.13	0.20	0.01	0.67	0.49	0.06	0.33	0.64
**NDVI65 ^25^**	1.18	2.13 *	0.02	0.04	1.24	0.09	0.06	0.02	0.07	0.01	0.15	0.07	0.01	0.08	0.22
**NDVI83 ^26^**	30.83 ***	7.45 ***	5.54 *	56.25 ***	3.86 ***	0.48	0.88	0.35	37.11 ***	0.71	0.28	0.15	0.16	0.49	0.49
**RWC ^27^**	858.81 ***	0.92	45.38 ***	1.47	1.05	0.48	0.36	0.56	0.53	0.08	0.41	1.47	3.92 *	0.56	0.68
**WW65 ^28^**	1.32	3.60 **	0.18	0.26	1.74	0.20	0.24	0.11	0.02	0.02	0.60	0.12	0.01	0.24	0.36
**DW65 ^29^**	407.65 ***	1.84	49.66 ***	34.36 ***	1.16	1.26	0.99	4.04	1.43	4.60	1.23	0.49	0.19	0.87	0.63
**WW91 ^30^**	202.57 ***	3.50	47.54 ***	26.68 ***	1.38	0.42	0.76	45.45 ***	6.93 *	7.22 *	0.73	0.54	3.26	1.19	0.08
**DW91 ^31^**	261.27 ***	3.35	39.82 ***	17.72 ***	1.48	0.34	0.65	40.35 ***	2.94	6.07 *	0.61	0.75	4.95	1.08	0.12

^1^ Days to heading; ^2^ days to flowering; ^3^ days to maturity; ^4^ grain yield; ^5^ thousand-grain weight; ^6^ seed number/main spike; ^7^ seed weight/main spike; ^8^ spikelet number/main spike; ^9^ basal sterile spikelet number/main spike; ^10^ apical sterile spikelet number/main spike; ^11^ seed length; ^12^ seed width; ^13^ plant height up to the flag leaf collar; ^14^ plant height up to the base of the ear; ^15^ plant height up to the tip of the ear; ^16^ ear length; ^17^ fertile tiller number; ^18^ flag leaf area; ^19^ SPAD value at Z45; ^20^ SPAD value at Z65; ^21^ SPAD value at Z77; ^22^ SPAD value at Z83; ^23^ SPAD value at Z85; ^24^ normalized difference vegetation index (NDVI) at Z45; ^25^ NDVI at Z65; ^26^ NDVI at Z83; ^27^ relative water content; ^28^ fresh biomass at Z65; ^29^ dry biomass at Z65; ^30^ fresh biomass at Z91; ^31^ dry biomass at Z91.

**Table 2:** Mean values of plant development, morphological and physiological traits and of yield components recorded for *QYld.idw-3B−− and QYld.idw-3B++* near-isogenic lines of spring durum wheat in rain-fed and well-watered treatments. *, ** and *** denote significant differences within treatments between the *QYld.idw-3B−− and QYld.idw-3B++* near-isogenic lines at the *p* < 0.05, 0.01 and 0.001 levels of probability, respectively (2013).

	**HD ^1^**		**FD ^2^**		**MD ^3^**		**SPAD45 ^4^**		**SPAD65 ^5^**		**SPAD77 ^6^**		**SPAD83 ^7^**		**SPAD85 ^8^**		**NDVI45 ^9^**		**NDVI65 ^10^**		**NDVI83 ^11^**		**AUSDC ^12^**		**AUVIC ^13^**		**FLA ^14^**	
	***W***	***NW***	***W***	***NW***	***W***	***NW***	***W***	***NW***	***W***	***NW***	***W***	***NW***	***W***	***NW***	***W***	***NW***	***W***	***NW***	***W***	***NW***	***W***	***NW***	***W***	***NW***	***W***	***NW***	***W***	***NW***
**NIL1−−**	150.56 ***	150.83	156 ***	156.36	194.72 **	193.27	42.10	41.16	48.30	48.48	37.41	30.78	29.32	21.21	10.92	9.80	0.3498	0.3546	0.5515	0.5502	0.3397	0.3279	1597.95	1441.53	14.86	15.31	27.65 **	26.22
**NIL++**	149.09	148.94	154.44	154.39	194.36	193.14	42.97	42.91	48.85	48.89	43.81	41.57	34.40 ***	26.95 **	10.72	10.73	0.3585	0.3563	0.5515	0.5532	0.3900 ***	0.3800*	1839.04 ***	1734.17 **	15.57	15.65	23.55	24.48
**LSD5%**	0.45	2.27	0.66	2.15	0.19	1.91	1.06	3.06	1.37	2.78	2.41	3.38	1.44	3.43	2.68	2.10	0.0082	0.0247	0.0044	0.0115	0.0179	0.0429	72.74	175.27	0.45	1.69	2.52	3.67
**LSD1%**	0.67	3.36	0.97	3.18	0.28	2.82	1.58	4.53	2.04	4.11	3.58	5.01	2.14	5.08	3.96	3.12	0.01223	0.0366	0.0065	0.0170	0.0265	0.0635	107.65	259.40	0.67	2.51	3.74	5.44
**LSD0.1%**	1.04	5.19	1.50	4.91	0.43	4.37	2.44	7.01	3.15	6.36	5.53	7.74	3.30	7.85	6.13	4.82	0.0189	0.0566	0.0101	0.0263	0.0409	0.0982	166.36	400.86	1.03	3.87	5.78	8.41
	**FTN ^15^**		**FLC ^16^**		**BE ^17^**		**TE ^18^**		**EL ^19^**		**GY ^20^**		**SKNM ^21^**		**SNM ^22^**		**SWM ^23^**		**BSM ^24^**		**ASM ^25^**		**TGW ^26^**		**SL ^27^**		**SW ^28^**	
	***W***	***NW***	***W***	***NW***	***W***	***NW***	***W***	***NW***	***W***	***NW***	***W***	***NW***	***W***	***NW***	***W***	***NW***	***W***	***NW***	***W***	***NW***	***W***	***NW***	***W***	***NW***	***W***	***NW***	***W***	***NW***
**NIL1−−**	370.59	378.34	40.59	40.90	55.68	54.14	61.79	59.85	5.93	5.65	3.02	2.67	13.54	13.51	35.25	34.83	1.88	1.44	0.7582	0.8586	0.5433	0.5419	35.43	43.63	7.01	6.99	2.89	3.06
**NIL++**	400.63 **	392.30 **	43.13 **	43.52 *	62.66 **	62.05 ***	69.33 ***	67.82 ***	6.21	5.81	3.42 ***	3.15 ***	13.42	13.32	37.52 **	37.15 ***	2.23 **	1.64 **	0.6176	0.6861	0.5433	0.5070	39.82 ***	46.50 **	7.01	7.02	3.05 ***	3.16 *
**LSD5%**	18.59	6.60	1.15	2.36	3.16	2.86	2.91	2.69	0.10	0.22	0.15	0.20	0.22	0.33	1.02	0.98	0.15	0.13	0.4992	0.3313	0.1889	0.1608	1.12	1.70	0.03	0.09	0.05	0.09
**LSD1%**	27.52	9.77	1.71	3.50	4.67	4.23	4.31	3.98	0.16	0.33	0.23	0.30	0.32	0.48	1.51	1.45	0.23	0.19	0.7388	0.4902	0.2796	0.2380	1.66	2.52	0.05	0.14	0.07	0.24
**LSD0.1%**	42.53	15.11	2.64	5.41	7.23	6.54	6.67	6.15	0.24	0.52	0.35	0.47	0.50	0.75	2.33	2.24	0.35	0.30	1.1417	0.7576	0.4321	0.3678	2.57	3.90	0.08	0.22	0.11	0.37
	**HL ^29^**		**P ^30^**		**G ^31^**		**Z ^32^**		**WW65 ^33^**		**WW91 ^34^**		**DW65 ^35^**		**DW91 ^36^**		**RWC ^37^**		**APX ^38^**		**GPX ^39^**		**PUT ^40^**		**SPD ^41^**		**SPN ^42^**	
	***W***	***NW***	***W***	***NW***	***W***	***NW***	***W***	***NW***	***W***	***NW***	***W***	***NW***	***W***	***NW***	***W***	***NW***	***W***	***NW***	***W***	***NW***	***W***	***NW***	***W***	***NW***	***W***	***NW***	***W***	***NW***
**NIL1−−**	76.74	82.54	17.31 ***	16.76	36.18 **	35.09	62.09	64.11	0.1444	0.1439	0.0340	0.0339	0.0195	0.0140	0.0240	0.0161	91.80	87.62	824.89	576.57	3040.49	4899.19	192.87	355.05	394.47	524.73	217.78	296.51
**NIL++**	78.93	82.57	16.16	17.07	34.01	35.39	61.34	65.91	0.1437	0.1442	0.0389 *	0.0380	0.0186	0.0161	0.0281*	0.0280	91.07	87.52	982.15	504.77	3438.56	4840.85	177.70	460.20*	419.43	572.83	305.50	342.78
**LSD5%**	1.54	1.36	0.45	1.39	1.17	2.36	1.01	2.87	0.0039	0.0054	0.0045	0.0094	0.0014	0.0039	0.0034	0.0069	1.75	2.88	282.90	78.58	1331.83	3291.57	86.88	91.89	24.13	171.95	113.38	59.48
**LSD1%**	2.28	2.01	0.67	2.06	1.73	3.50	1.49	4.25	0.0058	0.0080	0.0067	0.0139	0.0021	0.0058	0.0051	0.0102	2.59	4.26	519.22	144.23	2444.38	6041.20	159.46	168.66	44.30	315.60	208.10	109.05
**LSD0.1%**	3.52	3.11	1.04	3.19	2.67	5.41	2.31	6.56	0.0090	0.0124	0.0104	0.0215	0.0033	0.0090	0.0078	0.0158	4.01	6.58	1148.88	319.14	5408.61	13367.16	352.84	373.20	98.03	698.31	460.47	241.29

^1^ Days to heading; ^2^ days to flowering; ^3^ days to maturity; ^4^ SPAD value at Z45; ^5^ SPAD value at Z65; ^6^ SPAD value at Z77; ^7^ SPAD value at Z83; ^8^ SPAD value at Z85; ^9^ NDVI at Z45; ^10^ NDVI at Z65; ^11^ NDVI at Z83; ^12^ area under SPAD value decline curve; ^13^ area under vegetation index curve; ^14^ flag leaf area; ^15^ fertile tiller number; ^16^ plant height up to the flag leaf collar; ^17^ plant height up to the base of the ear; ^18^ plant height up to the tip of the ear; ^19^ ear length; ^20^ grain yield; ^21^ spikelet number/main spike; ^22^ seed number/main spike; ^23^ seed weight/main spike; ^24^ basal sterile spikelet number/main spike; ^25^ apical sterile spikelet number/main spike; ^26^ thousand-grain weight; ^27^ seed length; ^28^ seed width; ^29^ test weight; ^30^ protein content; ^31^ glutenin content; ^32^ Zeleny index; ^33^ fresh biomass at Z65; ^34^ fresh biomass at Z91; ^35^ dry biomass at Z65; ^36^ dry biomass at Z91; ^37^ relative water content; ^38^ ascorbate peroxidase; ^39^ guaiacol peroxidase; ^40^ putrescine; ^41^ spermidine; ^42^ days to spermine.

**Table 3:** Mean values of plant development, morphological and physiological traits and of yield components recorded for *QYld.idw-3B−− and QYld.idw-3B++* near-isogenic lines of spring durum wheat in rain-fed and well-watered treatments. *, ** and *** denote significant differences within treatments between the *QYld.idw-3B−− and QYld.idw-3B++* near-isogenic lines at the *p* < 0.05, 0.01 and 0.001 levels of probability, respectively (2014).

	**HD ^1^**		**FD ^2^**		**MD ^3^**		**SPAD45 ^4^**		**SPAD65 ^5^**		**SPAD77 ^6^**		**SPAD83 ^7^**		**SPAD85 ^8^**		**NDVI45 ^9^**		**NDVI65 ^10^**		**NDVI83 ^11^**		**AUSDC ^12^**		**AUVIC ^13^**	
	***W***	***NW***	***W***	***NW***	***W***	***NW***	***W***	***NW***	***W***	***NW***	***W***	***NW***	***W***	***NW***	***W***	***NW***	***W***	***NW***	***W***	***NW***	***W***	***NW***	***W***	***NW***	***W***	***NW***
**NIL1−−**	146.50	146.09	150.43	150.00	189.09	186.42	52.21	52.23	48.30	51.871875	46.03	41.92	29.45	21.49	12.13	9.19	0.3527	0.3522	0.5574	0.5557	0.3829	0.3700	2170.42	2020.28	24.89	22.14
**NIL++**	146.05	145.71	150	149.62	189.70	188.04	53.67	53.18	48.85	54.20 *	47.95	44.94	31.47	24.81 **	13.31	10.03	0.3527	0.3523	0.5557	0.5556	0.4000 **	0.3900 *	2229.15	2109.61 **	26.28 *	22.54
**LSD5%**	1.23	1.37	1.12	1.31	1.15	1.84	1.14	1.14	1.37	1.63	2.01	2.09	2.38	1.66	1.25	0.96	0.0032	0.0024	0.0047	0.0039	0.0087	0.0156	98.81	45.39	0.96	0.86
**LSD1%**	1.82	2.03	1.66	1.95	1.71	2.72	1.69	1.68	2.04	2.42	2.97	3.10	3.52	2.47	1.85	1.42	0.0048	0.0036	0.0070	0.0059	0.0129	0.0231	146.23	67.18	1.41	1.28
**LSD0.1%**	2.81	3.14	2.56	3.01	2.64	4.21	2.61	2.61	3.15	3.74	4.59	4.79	5.44	3.81	2.86	2.19	0.0074	0.0055	0.0108	0.0091	0.0199	0.0357	225.98	103.82	2.19	1.98
	**FLA ^14^**		**FTN ^15^**		**FLC ^16^**		**BE ^17^**		**TE ^18^**		**EL ^19^**		**GY ^20^**		**SKNM ^21^**		**SNM ^22^**		**SWM ^23^**		**BSM ^24^**		**ASM ^25^**		**TGW ^26^**	
	***W***	***NW***	***W***	***NW***	***W***	***NW***	***W***	***NW***	***W***	***NW***	***W***	***NW***	***W***	***NW***	***W***	***NW***	***W***	***NW***	***W***	***NW***	***W***	***NW***	***W***	***NW***	***W***	***NW***
**NIL1−−**	26.41	27.37	361.31	362.78	51.43	48.33	65.57	63.97	73.59	70.55	6.73	6.31	3.59	2.58	15.25	14.98	44.71	44.26	2.11	1.71	0.6125	0.5146	0.4817	0.8330	37.67	38.83
**NIL++**	28.02	27.49	423.81 **	407.77 ***	52.84	50.47 *	73.50 **	68.67 *	80.00 **	76.02 **	7.11 **	6.64 ***	4.40 **	3.22 ***	15.21	14.89	46.32 **	45.75 **	2.51 ***	1.97 **	0.5981	0.7326	0.4871	0.5387	40.82 **	40.93 ***
**LSD5%**	2.59	1.08	32.77	15.15	3.72	1.54	3.49	3.78	4.19	3.46	0.16	0.14	0.45	0.22	0.19	0.31	1.07	1.00	0.09	0.13	0.2308	0.4637	0.1675	0.6190	1.60	0.52
**LSD1%**	3.84	1.60	48.50	22.41	5.50	2.28	5.17	5.60	6.21	5.13	0.24	0.20	0.67	0.33	0.28	0.46	1.59	1.48	0.14	0.20	0.3415	0.6863	0.2479	0.9161	2.37	0.77
**LSD0.1%**	5.94	2.49	74.96	34.64	8.50	3.52	7.99	8.66	9.59	7.92	0.38	0.31	1.04	0.51	0.44	0.72	2.46	2.28	0.22	0.31	0.5278	1.0606	0.3831	1.4158	3.67	1.19
	**SL ^27^**		**SW ^28^**		**HL ^29^**		**P ^30^**		**G ^31^**		**Z ^32^**		**WW65 ^33^**		**WW91 ^34^**		**DW65 ^35^**		**DW91 ^36^**		**RWC ^37^**					
	***W***	***NW***	***W***	***NW***	***W***	***NW***	***W***	***NW***	***W***	***NW***	***W***	***NW***	***W***	***NW***	***W***	***NW***	***W***	***NW***	***W***	***NW***	***W***	***NW***				
**NIL1−−**	7.05	7.02	2.97	2.87	78.37	79.64	18.18	17.75	38.13	36.98	65.24 *	63.25	0.1399	0.1408	0.0735	0.0456	0.0358	0.0297	0.0614	0.0371	73.20	68.58				
**NIL++**	7.01	7.02	2.97	2.88	78.72	80.24	17.44	17.82	36.89	37.16	64.00	63.67	0.1567 ***	0.1525	0.0685	0.0503	0.03693	0.0283	0.0590	0.0428	73.07	67.80				
**LSD5%**	0.0601	0.0344	0.0502	0.0532	2.08	1.02	1.08	0.8754	1.58	1.35	1.19	1.03	0.0062	0.0084	0.0102	0.0094	0.0052	0.0036	0.0107	0.0088	2.30	4.11				
**LSD1%**	0.0890	0.0509	0.0743	0.0788	3.08	1.51	1.60	1.2956	2.34	1.99	1.76	1.52	0.0092	0.0124	0.0151	0.0139	0.0077	0.0053	0.0159	0.0131	3.40	6.08				
**LSD0.1%**	0.1376	0.0786	0.1149	0.1218	4.76	2.33	2.48	2.0022	3.63	3.08	2.73	2.35	0.0142	0.0192	0.0234	0.0216	0.0120	0.0083	0.0246	0.0203	5.26	9.40				

^1^ Days to heading; ^2^ days to flowering; ^3^ days to maturity; ^4^ SPAD value at Z45; ^5^ SPAD value at Z65; ^6^ SPAD value at Z77; ^7^ SPAD value at Z83; ^8^ SPAD value at Z85; ^9^ NDVI at Z45; ^10^ NDVI at Z65; ^11^ NDVI at Z83; ^12^ area under SPAD value decline curve; ^13^ area under vegetation index curve; ^14^ flag leaf area; ^15^ fertile tiller number; ^16^ plant height up to the flag leaf collar; ^17^ plant height up to the base of the ear; ^18^ plant height up to the tip of the ear; ^19^ ear length; ^20^ grain yield; ^21^ spikelet number/main spike; ^22^ seed number/main spike; ^23^ seed weight/main spike; ^24^ basal sterile spikelet number/main spike; ^25^ apical sterile spikelet number/main spike; ^26^ thousand-grain weight; ^27^ seed length; ^28^ seed width; ^29^ test weight; ^30^ protein content; ^31^ glutenin content; ^32^ Zeleny index; ^33^ fresh biomass at Z65; ^34^ fresh biomass at Z91; ^35^ dry biomass at Z65; ^36^ dry biomass at Z91; ^37^ relative water content.

**Table 4:** Correlations between morphological and physiological parameters and yield components in the rain-fed treatment. *, **, *** significant at the *p* < 0.05, 0.01 and 0.001 probability levels, respectively.

Traits	Simple Regression between the Traits (R^2^)				
	GY ^1^	TGW ^2^	SNM ^3^	SWM ^4^	SW ^5^
	Grand mean for years and treatments				
NDVI45 ^6^					
NDVI65 ^7^					
NDVI83 ^8^	38.16 ***		18.29 *	45.78 ***	
SPAD45 ^9^					
SPAD65 ^10^	13.33 *	16.49 *		27.78 ***	25.26 **
SPAD77 ^11^	32.73 ***		38.41 ***	38.98 ***	
SPAD83 ^12^	44.38 ***			21.38 ***	
SPAD85 ^13^	16.51 *				
FLC ^14^	15.27 *	33.11 ***			16.00 *
BE ^15^	33.35 ***				
TE ^16^	34.04 ***	12.81 *			
EL ^17^		37.42 ***			34.36 ***
FTN ^18^	40.41 ***		48.87 ***	22.79 ***	
FLA ^19^			14.65 *		
WW65 ^20^	17.55 *	18.89 *		37.34 ***	30.37 **
DW65 ^21^	12.74 *	31.45 ***		39.07 ***	15.08 *

^1^ Grain yield; ^2^ thousand-grain weight; ^3^ seed number/main spike; ^4^ seed weight/main spike; ^5^ seed width; ^6^ NDVI at Z45; ^7^ NDVI at Z65; ^8^ NDVI value at Z83; ^9^ SPAD at Z45; ^10^ SPAD at Z65; ^11^ SPAD value at Z77; ^12^ SPAD value at Z83; ^13^ SPAD value at Z85; ^14^ plant height up to the flag leaf collar; ^15^ plant height up to the base of the ear; ^16^ plant height up to the tip of the ear; ^17^ ear length; ^18^ fertile tiller number; ^19^ flag leaf area; ^20^ fresh biomass at Z65; ^21^ dry biomass at Z65.

## Supplementary Materials

Supplementary Materials can be found at https://www.mdpi.com/1422-0067/22/4/2053/s1.

## Abbreviations

AUSDC	area under SPAD value decline curve
AUVIC	area under vegetation index curve
HD	heading date
SPAD	SPAD unit
NDVI	normalized difference vegetation index
GY	grain yield (t/ha)
FW	biomass fresh weight (kg)
DW	biomass dry weight (kg)
HL	test weight (HL)
FLC	plant height up to the flag leaf collar (cm)
BE	plant height up to the base of the ear (cm)
TE	plant height up to the tip of the ear (cm)
FTN	fertile tiller number (pcs./m2)
SNM	seed number/main spike
SWM	seed weight/main spike (g)
SW	seed width (cm)
SL	seed length (cm)
TGW	thousand-grain weight (g)
RWC	relative water content (%)
APX	ascorbate peroxidase (nkatal g-1 DW)
GPX	guaiacol peroxidase (nkatal g-1 DW)
PUT	putrescine (mg g-1 DW)
SPD	spermidine (mg g-1 DW)
SPN	spermine (mg g-1 DW)
Z	Zadoks scale
